# Supplementary material for: Germline variant burden in cancer genes correlates with age at diagnosis and somatic mutation burden
Source: Nat Commun. 2020 May 15;11:2438. doi: 10.1038/s41467-020-16293-7 (PMC7228928; doi:10.1038/s41467-020-16293-7)
Supplement: Supplementary file 1 — Supplementary Information [file 41467_2020_16293_MOESM1_ESM.docx]

**Supplementary Information**

Germline variant burden in cancer genes correlates with age at diagnosis and somatic mutation burden

Qing et. al.

**Supplementary Table 1.** TCGA samples used in this analysis

| **NO.** | **Cancers** | **Abbreviation** | **# European Adults** | **Total Samples** |
| --- | --- | --- | --- | --- |
| 1 | Glioblastoma multiforme | GBM | 333 | 393 |
| 2 | Ovarian serous cystadenocarcinoma | OV | 325 | 412 |
| 3 | Lung adenocarcinoma | LUAD | 387 | 518 |
| 4 | Lung squamous cell carcinoma | LUSC | 340 | 499 |
| 5 | Prostate adenocarcinoma | PRAD | 408 | 498 |
| 6 | Uterine Corpus Endometrial Carcinoma | UCEC | 358 | 543 |
| 7 | Bladder Urothelial Carcinoma | BLCA | 326 | 412 |
| 8 | Testicular Germ Cell Tumors | TGCT | 109 | 134 |
| 9 | Esophageal carcinoma | ESCA | 113 | 184 |
| 10 | Pancreatic adenocarcinoma | PAAD | 152 | 185 |
| 11 | Kidney renal papillary cell carcinoma | KIRP | 199 | 289 |
| 12 | Liver hepatocellular carcinoma | LIHC | 178 | 375 |
| 13 | Cervical squamous cell carcinoma and endocervical adenocarcinoma | CESC | 190 | 305 |
| 14 | Sarcoma | SARC | 208 | 255 |
| 15 | Breast invasive carcinoma | BRCA | 745 | 1076 |
| 16 | Thymoma | THYM | 99 | 123 |
| 17 | Mesothelioma | MESO | 80 | 82 |
| 18 | Colon adenocarcinoma | COAD | 205 | 419 |
| 19 | Stomach adenocarcinoma | STAD | 274 | 443 |
| 20 | Skin Cutaneous Melanoma | SKCM | 432 | 470 |
| 21 | Cholangiocarcinoma | CHOL | 31 | 45 |
| 22 | Kidney renal clear cell carcinoma | KIRC | 302 | 387 |
| 23 | Thyroid carcinoma | THCA | 316 | 499 |
| 24 | Head and Neck squamous cell carcinoma | HNSC | 434 | 526 |
| 25 | Rectum adenocarcinoma | READ | 79 | 145 |
| 26 | Brain Lower Grade Glioma | LGG | 467 | 515 |
| 27 | Kidney Chromophobe | KICH | 57 | 66 |
| 28 | Uterine Carcinosarcoma | UCS | 44 | 57 |
| 29 | Adrenocortical carcinoma | ACC | 75 | 92 |
| 30 | Pheochromocytoma and Paraganglioma | PCPG | 147 | 179 |
| 31 | Uveal Melanoma | UVM | 55 | 80 |


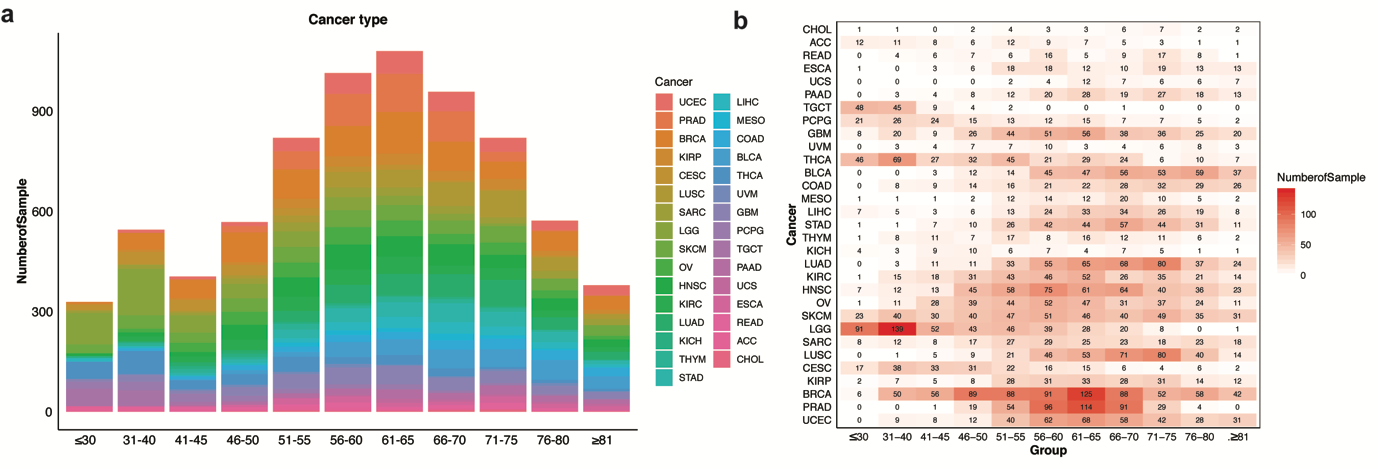


**Supplementary Figure 1. The distribution of cancer types in each age group. (a)** The number of samples in eleven age groups. Color represents cancer type. **(b)** The cancer-type distribution in eleven age groups.


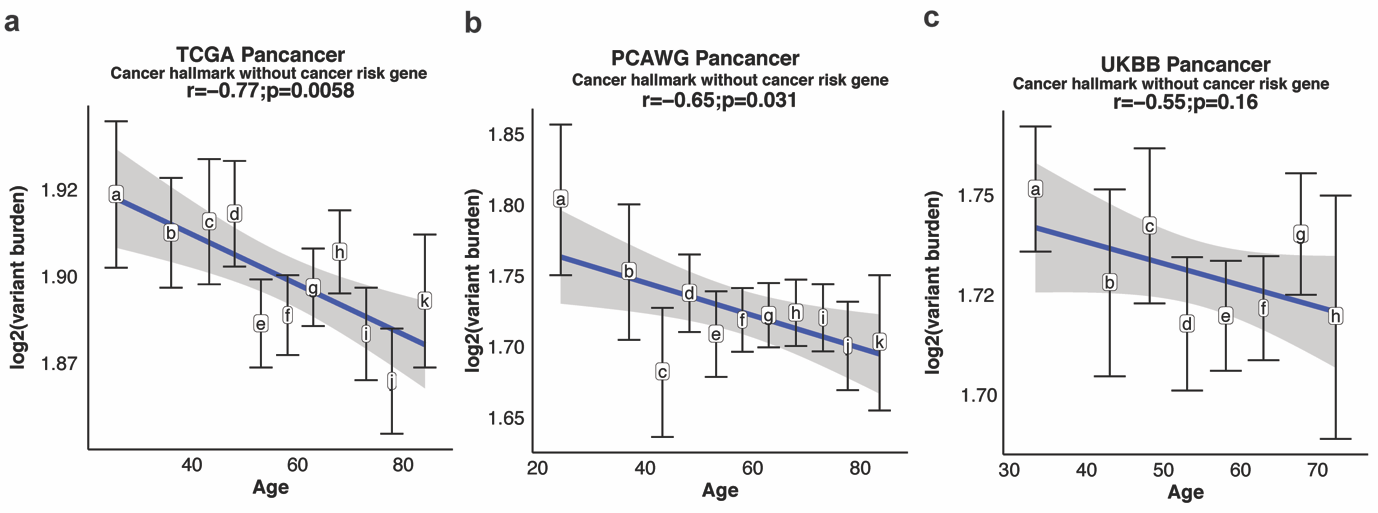


**Supplementary Figure 2**. **Correlations between age and the average gHFI burden in cancer hallmark genes without including cancer predisposition genes** (n = 1,508). **(a)** TCGA (n = 7,468 cases) **(b)** PCWAG (n = 1,487 cases) **(c)** UK BioBank (n = 7,198 cases).


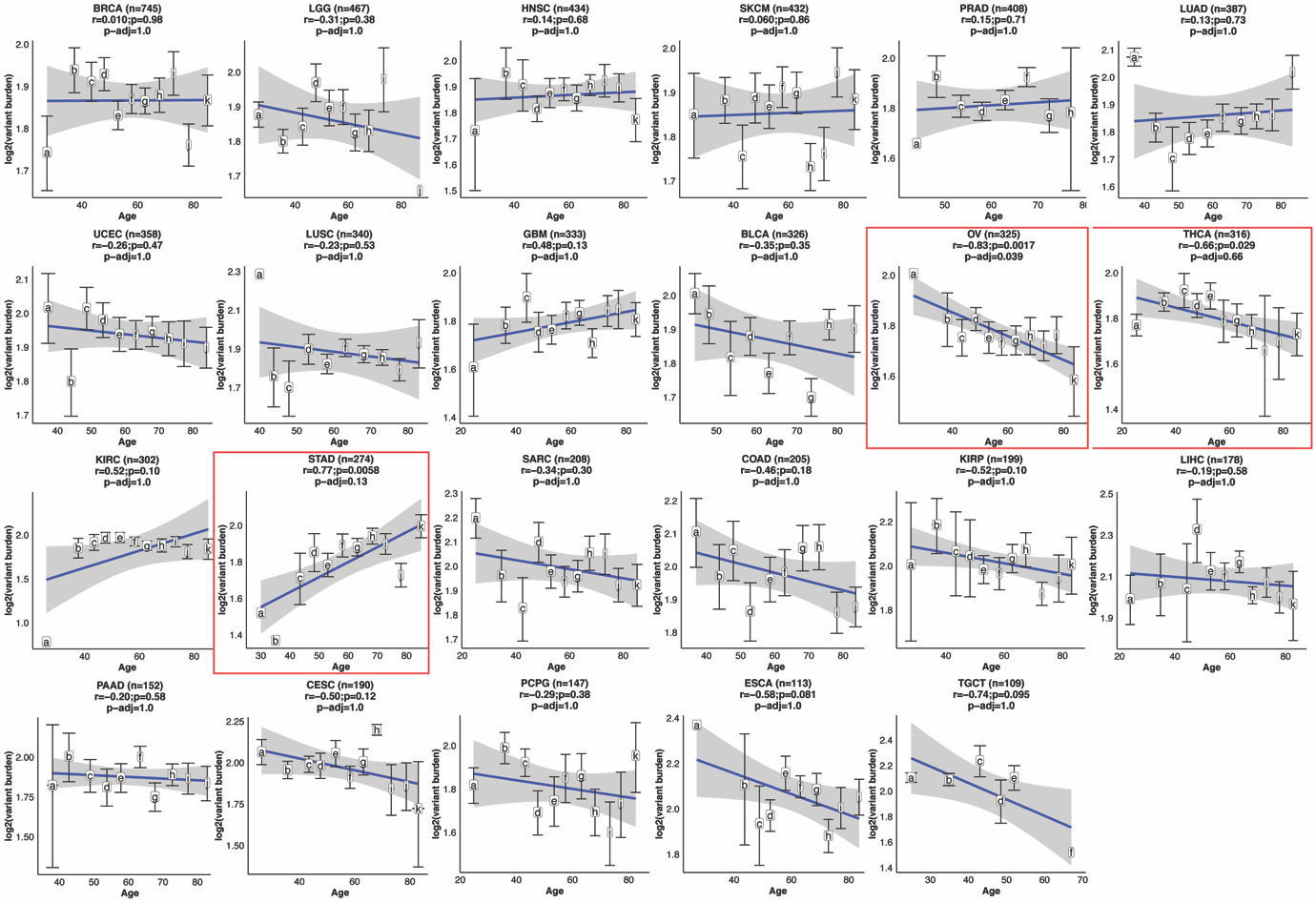


**Supplementary Figure 3**. **Correlation between gHFI variant burden and age in cancer hallmark genes excluding 50 NCCN hereditary cancer genes.** **(a)** Average variant burden for 11 age intervals (the tags a-k correspond to ages ≤ 30, 31-40, 41-45, 46-50, 51-55, 56-60, 61-65, 66-70, 71-75, 76-80, and ≥ 81.). **(b)** Correlation between average variant burden and average mutation burden in cancer hallmark genes excluding 50 NCCN hereditary cancer genes across age. Tags a-k indicate age intervals corresponding to ages ≤30, 31-40, 41-45, 46-50, 51-55, 56-60, 61-65, 66-70, 71-75, 76-80, and ≥81 years. The y-axes correspond to log_2_ transformed variant/mutation burden. Error bars represent standard error. The r represents Pearson correlation coefficient. Spearman's Rho test (two-sided) was used to generate the p-value to measure the strength of correlation coefficient. The p-adj represents adjusted p value using Holm’s method.


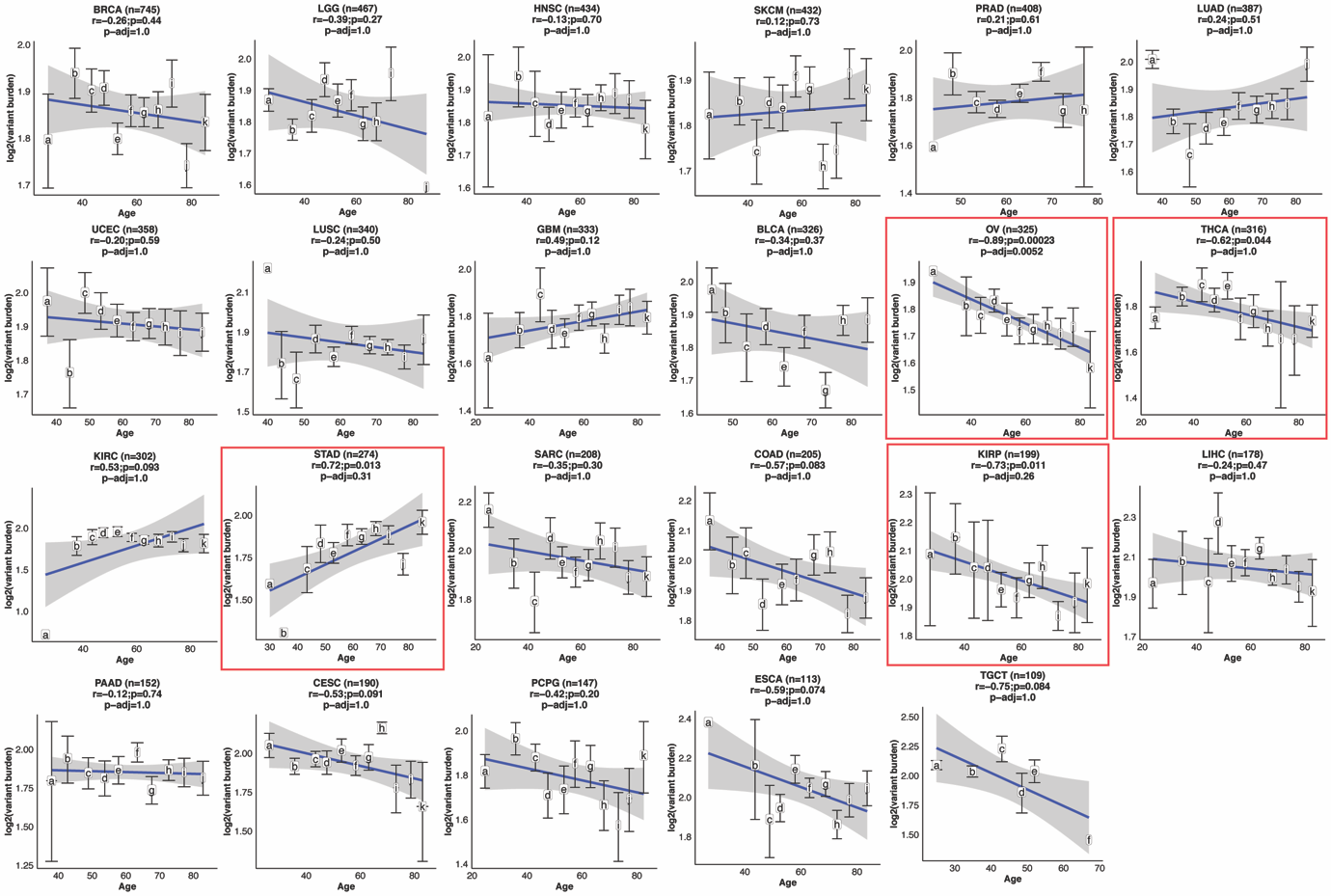


**Supplementary Figure 4. Correlations between average gHFI variant burden and age at cancer diagnosis in cancer types**. The red boxes highlight the significant associations with *P* < 0.05. P-values are not adjusted for the multiple group comparisons. The tags a-k correspond to ages ≤30, 31-40, 41-45, 46-50, 51-55, 56-60, 61-65, 66-70, 71-75, 76-80, and ≥81. Error bars represent standard error. The r represents Pearson correlation coefficient. Spearman's Rho test (two-sided) was used to generate the p-value to measure the strength of correlation coefficient. The p-adj represents adjusted p value using Holm’s method.


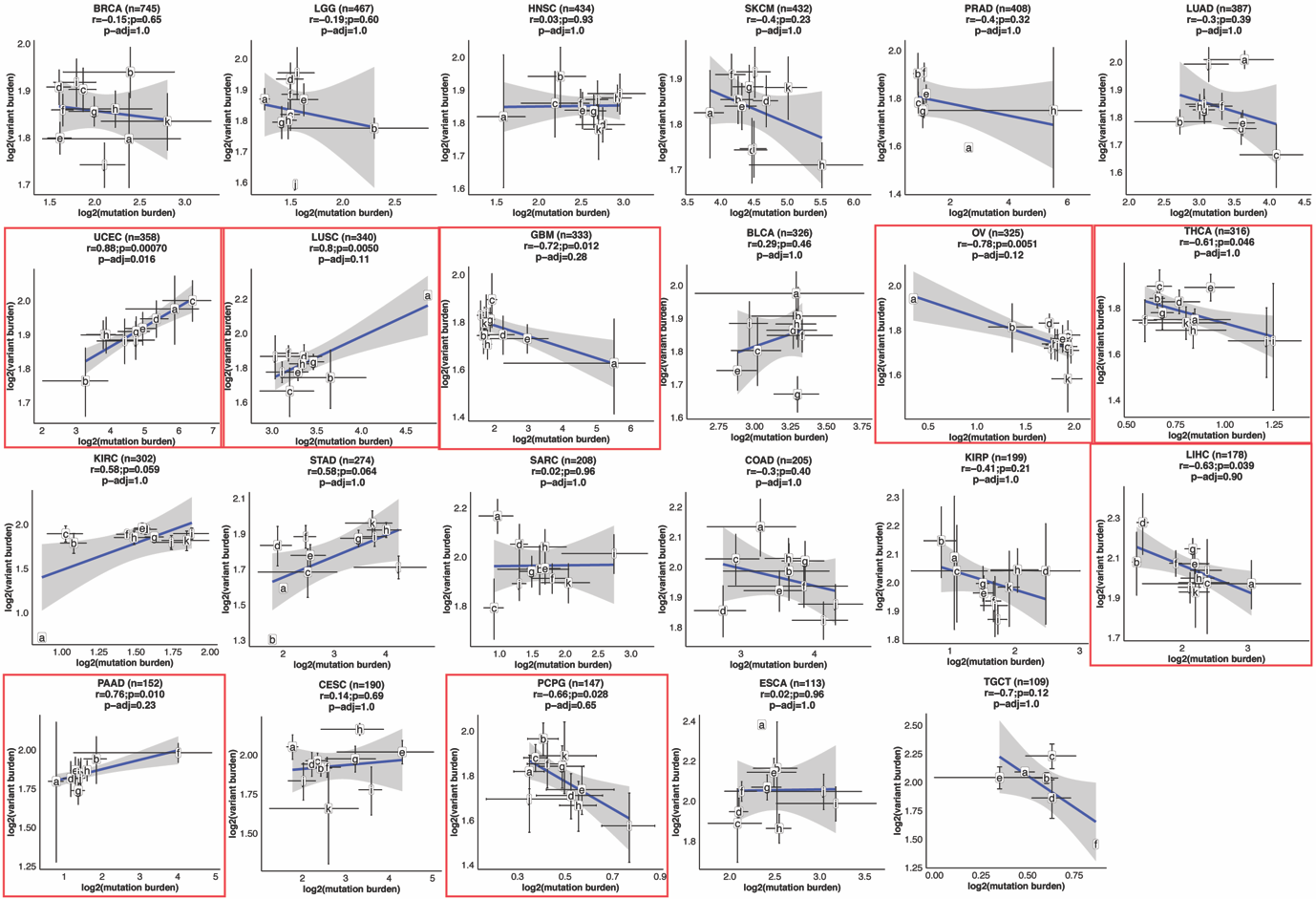


**Supplementary Figure 5. Log-log age interval scatter plots between average germline high-functional variant burden versus the mutation burden by age intervals in each TCGA cancer type.** The red boxes highlight the significant associations with *P* < 0.05. P-values are not adjusted for the multiple group comparisons. The tags a-k correspond to ages ≤30, 31-40, 41-45, 46-50, 51-55, 56-60, 61-65, 66-70, 71-75, 76-80, and ≥81. Error bars represent standard error. The r represents Pearson correlation coefficient. Spearman's Rho test (two-sided) was used to generate the p-value to measure the strength of correlation coefficient. The p-adj represents adjusted p value using Holm’s method.


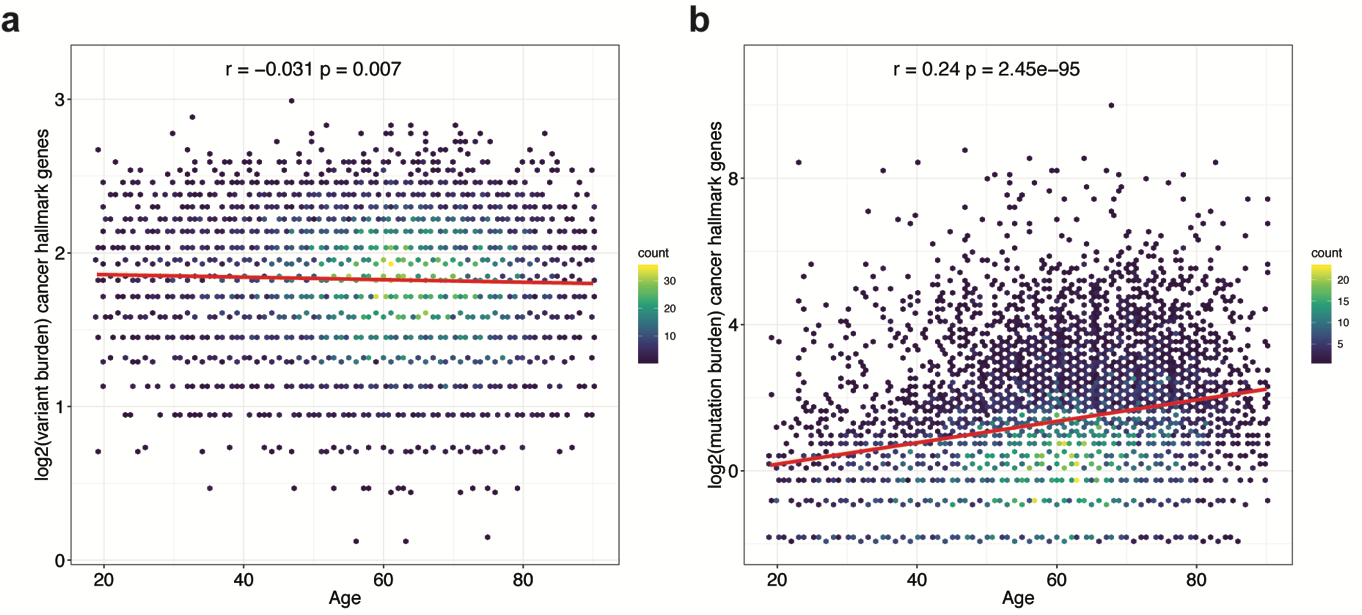


**Supplementary Figure 6.** **Density plots**. Age at diagnosis and (**a**) germline high-functional variant, and (**b**) somatic mutations in the TCGA. Each dot represents an individual; color scheme indicates increasing sample size in each dot from dark blue to yellow (some dots are overlapped), red line shows overall correlation fit.


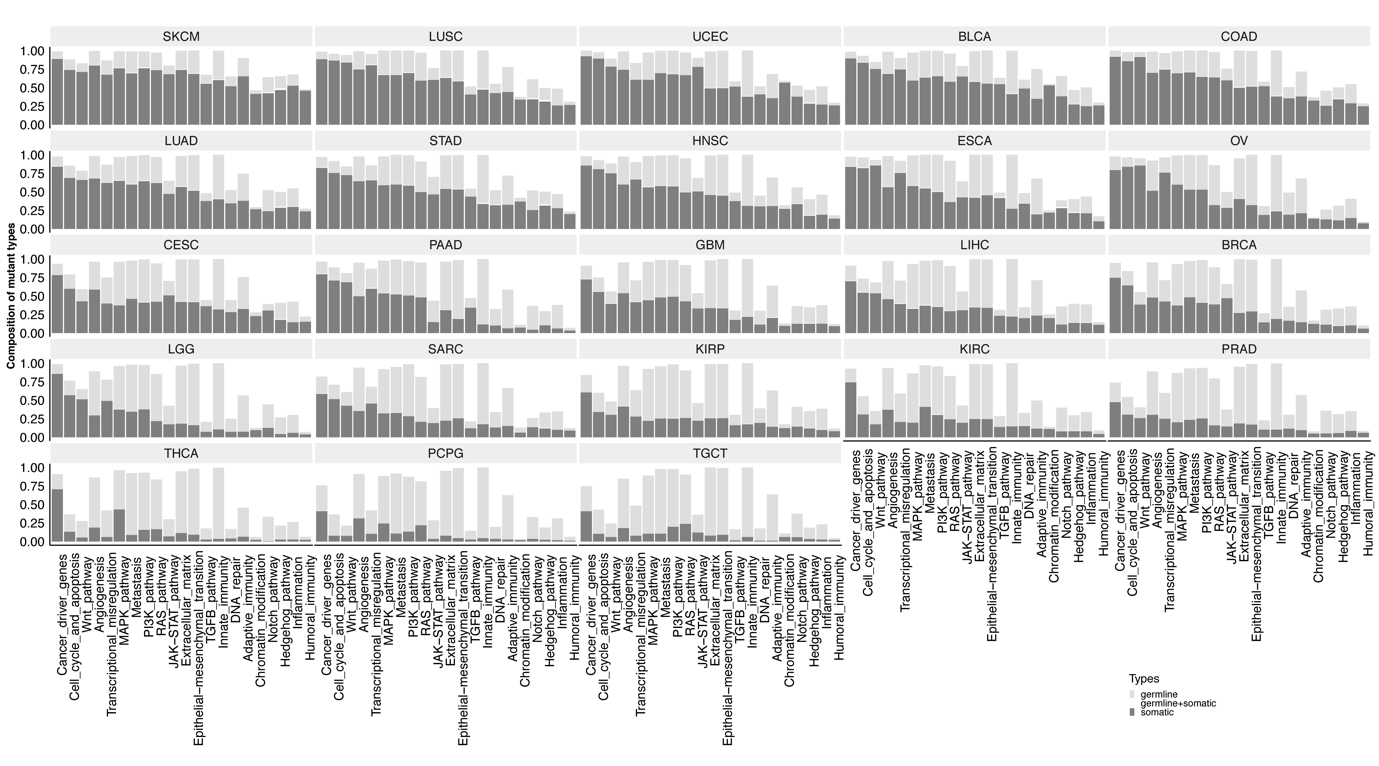


**Supplementary Figure 7**. **Average fractions of germline variants and somatic mutations across affected genes in 21 cancer hallmark pathways.** The average fractions of altered genes by source of alteration (germline versus somatic) is shown separately for each 23 cancer types in the TCGA.
