# Supplementary material for: Germline variant burden in cancer genes correlates with age at diagnosis and somatic mutation burden
Source: Nat Commun. 2020 May 15;11:2438. doi: 10.1038/s41467-020-16293-7 (PMC7228928; doi:10.1038/s41467-020-16293-7)
Supplement: Supplementary file 3 — Description of Additional Supplementary Information [file 41467_2020_16293_MOESM3_ESM.docx]

**Description of Additional Supplementary Files**

**File name:** Supplementary Data 1

**Description:** NanoString cancer hallmark pathways and member genes

**File name:** Supplementary Data 2

**Description:** NCCN Hereditary Cancer Genes
